# The effect of Meropenem stewardship at Prof Ngoerah Hospital on the prevalence of carbapenem resistant *Acinetobacter baumannii*, Carbapenem-resistant *Pseudomonas aeruginosa* and the cost of purchasing meropenem

**DOI:** 10.1017/ash.2025.87

**Published:** 2025-09-03

**Authors:** Made Susila Utama, Reza Andriani, Wayan Suranadi, Dwi Fatmawati

**Affiliations:** Antimicrobial Stewardship Team at Prof. Ngoerah Hospital, Denpasar, Bali

## Abstract

**Background:** Irrational use of antibiotics will trigger anti-microbial resistance which is a threat to health problems now and in the future. The use of meropenem is often the last choice in using antibiotics without undergoing microbiological examination (culture). The rational use of meropenem is expected to reduce resistant microbes as well as reducing hospital costs. Anti-microbial stewardship at Prof Ngoerah General Hospital began to be implemented at the end of 2020, and evaluation of the implementation of antimicrobial stewardship is required. **Objective:** The aim of this study was to determine the effect of meropenem stewardship on the prevalence of Carbapenem-resistant Acinetobacter Baumannii, Carbapenem-resistant Pseudomonas Aeruginosa and evaluate meropenem cost of purcashing. Retrospective cohort study from medical records, pharmacy records and microbiology data from microbiology from 2020 to 2023 was taken. Data is presented in the form of tables and graphs. **Results:** Before antimicrobial stewardship was implemented, in 2020 the prevalence of Carbapenem-resistant Acinetobacter Baumannii reached 69.1% and began to decline in 2021 by 52.35%, in 2022 it became 43.8% and 44.3% in 2023, respectively. The prevalence of Carbapenem-resistant Pseudomonas Aeruginosa also decreased, in 2020 was 31.6% to 27.3% in 2021, in 2022 it fell again to 24.8% and in 2023 only 18.4%. The cost of purchasing meropenem at Prof Ngoerah hospital before implementing antimicobial stewardship in 2020 was IDR 229,905,300,- decreasing to IDR 94,156,920,- in 2021, IDR 98,025,255 in 2022 and in 2023 IDR. 97,147,335,-, respectively. **Conclusions:** Meropenem stewardship at Prof. Ngoerah Hospital reduces the prevalence of Carbapenem-resistant Acinetobacter Baumannii and Carbapenem-resistant Pseudomonas Aeruginosa. Meropenem stewardship also reduces hospital costs in purchasing antibiotics.

